# Polydeoxynucleotide-Loaded Visible Light Photo-Crosslinked Gelatin Methacrylate Hydrogel: Approach to Accelerating Cartilage Regeneration

**DOI:** 10.3390/gels11010042

**Published:** 2025-01-07

**Authors:** Sunjae Park, Youngjun Son, Jonggyu Park, Soyoon Lee, Na-Hyeon Kim, Se-Na Jang, Tae-Woong Kang, Jeong-Eun Song, Gilson Khang

**Affiliations:** 1 Department of Polymer Nano Science & Technology and Polymer Materials Fusion Research Center, Jeonbuk National University, 567 Baekje-daero, Deokjin-gu, Jeonju-si 54896, Jeonbuk, Republic of Korea; sunjaepark@jbnu.ac.kr (S.P.); spring19963@jbnu.ac.kr (Y.S.); oort14@jbnu.ac.kr (J.P.); dlthdbs0401@jbnu.ac.kr (S.L.); skgus0316@naver.com (N.-H.K.); jsn819@jbnu.ac.kr (S.-N.J.); taewoongkang@jbnu.ac.kr (T.-W.K.); songje@jbnu.ac.kr (J.-E.S.); 2 Department of Bionanotechnology and Bio-Convergence Engineering, Jeonbuk National University, 567 Baekje-daero, Deokjin-gu, Jeonju-si 54896, Jeonbuk, Republic of Korea; 3 Department of Orthopaedic & Traumatology, Airlangga University, Jl. Airlangga No. 4–6, Airlangga, Kec. Gubeng, Kota SBY, Surabaya 60115, Jawa Timur, Indonesia

**Keywords:** cartilage regeneration, GelMA hydrogel, PDRN, visible light crosslinking, biocompatibility, tissue engineering

## Abstract

Articular cartilage faces challenges in self-repair due to the lack of blood vessels and limited chondrocyte concentration. Polydeoxyribonucleotide (PDRN) shows promise for promoting chondrocyte growth and cartilage regeneration, but its delivery has been limited to injections. Continuous PDRN delivery is crucial for effective cartilage regeneration. This study explores using gelatin methacrylate (gelMA) hydrogel, crosslinked with visible light and riboflavin 5′-phosphate sodium (RF) as a photoinitiator, for sustained PDRN release. GelMA hydrogel’s synthesis was confirmed through spectrophotometric techniques, demonstrating successful methacrylate group incorporation. PDRN-loaded gelMA hydrogels displayed varying pore sizes, swelling ratios, degradation rates, and mechanical properties based on gelMA concentration. They showed sustained PDRN release and biocompatibility, with the 14% gelMA-PDRN composition performing best. Glycosaminoglycan (GAG) activity was higher in PDRN-loaded hydrogels, indicating a positive effect on cartilage formation. RT-PCR analysis revealed increased expression of cartilage-specific genes (COL2, SOX9, AGG) in gelMA-PDRN. Histological assessments in a rabbit cartilage defect model demonstrated superior regenerative effects of gelMA-PDRN hydrogels. This study highlights the potential of gelMA-PDRN hydrogels in cartilage tissue engineering, providing a promising approach for effective cartilage regeneration.

## 1. Introduction

Articular cartilage is a specialized tissue with an excellent capacity to bear weight and reduce friction, allowing smooth joint movement. It comprises chondrocytes sparsely embedded in the extracellular matrix (ECM). However, its intrinsic regenerative capacity is limited due to the absence of blood vessels and the low density of chondrocytes [1,2]. The lack of blood vessels restricts the supply of nutrients and cells necessary for regenerating damaged tissue [3].

Existing surgical treatments, including arthroscopic debridement, cartilage transplantation, tibial/fibular osteotomy, and artificial joint replacement, have significant drawbacks such as high costs, limited donor availability, and the risk of autoimmune rejection [4,5,6,7,8]. Research suggests that polydeoxyribonucleotide (PDRN) can stimulate chondrocyte proliferation and enhance cartilage tissue regeneration. Derived from the sperm cells of salmon, PDRN consists of short DNA strands with 50–2000 base pairs [9]. Preclinical and clinical studies confirm the therapeutic potential of PDRN in the treatment of defects of cartilage [10,11]. PDRN facilitates effective cartilage regeneration by inhibiting proteoglycan degradation and reducing the expression of the MMP13 gene, which is responsible for cartilage collagen breakdown [12,13,14]. However, PDRN injections have limitations, such as uncontrolled release profiles, insufficient in situ dispersion, and limited sustained efficacy. To ensure prolonged release, this study aims to deliver PDRN using a hydrogel formulation.

Hydrogels are complex systems composed of a three-dimensional network of polymer chains and water, which occupies the spaces between macromolecules [15]. Due to their cartilage-like properties, hydrogels are being explored as potential treatments for cartilage abnormalities [16,17,18,19]. Table 1 presents a comparative analysis of various biomaterials used for cartilage regeneration scaffolds, highlighting their biocompatibility, biodegradability, gelation processes, hydrophilic/hydrophobic properties, and potential for cartilage regeneration.

Gelatin methacrylate (GelMA) is a gelatin derivative modified with methacrylic anhydride (MA). GelMA retains the temperature-sensitive gelation behavior of gelatin, with amino groups substituted by methacrylate groups through MA. GelMA also acquires photo-crosslinking characteristics and maintains the arginine-glycine-aspartate (RGD) sequence for cell adhesion and the matrix metalloproteinases (MMP) sequence for cell attachment [20,21,22,23]. Photo-initiated crosslinking with light exposure in the presence of a photoinitiator endows gelMA with exceptional thermal stability and mechanical strength, surpassing the limitations of gelatin. Therefore, in this study, gelMA concentrations of 12%, 14%, and 16% were selected to optimize the mechanical strength and biological compatibility of the hydrogel. These concentrations were determined to provide suitable cell adhesion and mechanical strength for cartilage regeneration based on previous studies and initial preliminary experiments [24,25,26].

**Table 1:** Comparative analysis of biomaterials for cartilage regeneration scaffolds.

	Gelatin Methacrylate (GelMA)	Chitosan	Polyethlene Glycol (PEG)	Polycaprolactone (PCL)	Polylactic Acid (PLA)
Biocompatibility	Excellent; supports cell adhesion and growth	High; promotes cell proliferation	Good; supports cell attachment and growth	Good; supports cell attachment and growth	Good; widely used in medical applications
Biodegradability	Biodegradable; rate adjustable by crosslinking	Biodegradable; consistent degradation profile	Biodegradable; rate adjustable by molecular weight	Slowly biodegradable; suitable for long-term applications	Biodegradable; rate adjustable by copolymer composition
Gelation Process	UV or visible light-induced crosslinking	Ionic crosslinking or temperature change	Chemical crosslinking or photopolymerization	Thermal processing or solvent casting	Thermal processing or solvent casting
Hydrophilic/ Hydrophobic	High water retention capacity	High water retention and good swelling properties	High water retention; non-fouling surface	Lower water retention; hydrophobic	Lower water retention; hydrophobic
Potential for Cartilage Regeneration	Promotes chondrogenesis with growth factors	Promotes chondrogenesis, especially in composites	Useful as a hydrogel; often combined with other materials	Excellent for load-bearing scaffolds; supports chondrogenesis	Promotes chondrogenesis; often used in combination with other materials
References	[8,27]	[28,29,30]	[31,32,33]	[34,35,36]	[37,38]

Most photoinitiators operate within the ultraviolet (UV) wavelength range (250–400 nm), but UV exposure can cause endogenous oxidative damage to DNA and exhibit cytotoxic effects [39]. Recent advancements have introduced near-infrared (NIR)-based photoinitiators for tissue regeneration, offering a promising alternative due to their deeper tissue penetration and reduced photodamage [40]. NIR photoinitiators generally have lower photoinitiation efficiency compared to UV and visible light photoinitiators. This means that they might require higher intensities or longer exposure times to achieve the same degree of polymerization [41]. In contrast, visible-light photoinitiators improve cell compatibility and reduce cell damage risk due to the absence of heat generation. Visible light, with longer wavelengths, penetrates tissues more effectively, leading to increased treatment depth [42,43]. Riboflavin 5′-phosphate sodium (RF), a derivative of vitamin B2, was used as a photoinitiator for constructing hydrogels crosslinked with visible light. RF undergoes reversible reduction and oxidation processes, involving the donation and acceptance of electron pairs, which prompts crosslinking through radical formation during redox reactions [44].

A fundamental challenge in cartilage tissue engineering is to formulate a comprehensive solution that combines favorable material characteristics with proper mechanical strength. In this study, gelMA concentrations of 12%, 14%, and 16% were applied to optimize the sustained release of PDRN and enhance cartilage regeneration. These concentrations were selected based on initial experimental results, considering the mechanical strength and cell compatibility of gelMA. This study aims to develop a biocompatible, visible light-crosslinked gelMA hydrogel incorporating PDRN, specifically tailored for effective cartilage regeneration. We evaluated the physicochemical properties of the hydrogel and the release profiles of PDRN across varying gelMA concentrations. Through in vitro analyses, we identified the optimal composition for promoting cartilage regeneration. Finally, the in vivo assessment of the PDRN-loaded hydrogel scaffold was conducted using a rabbit cartilage defect model to explore its potential for robust cartilage regeneration.

## 2. Results and Discussion

### 2.1. GelMA Hydrogel Fabrication and Charaterization

**Figure 1:**
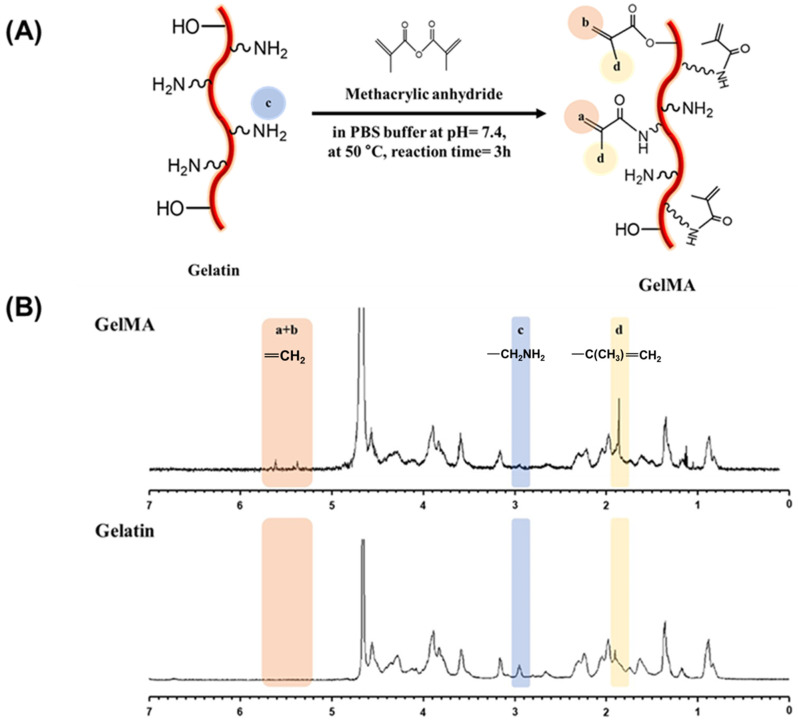
Schematic representation shows (**A**) Synthesis of gelMA with addition of methacrylic anhydride to gelatin (**B**) ^1^H NMR spectra of gelatin and gelMA demonstrating successful functionalization.

The gelMA used in this study was prepared according to a previously reported method [45]. Our initial aim was to analyze the chemical composition of the modified gelatin using spectrophotometric techniques. ^1^H-NMR spectroscopy is extensively employed for the purpose of chemical identification, as well as the analysis of functional groups and conformational changes in chemical processes. When comparing the NMR spectra of gelatin and gelMA (Figure 1), new peaks at 5.4 ppm and 5.6 ppm, absent in gelatin, are observed in gelMA. These correspond to the acrylic protons of the methacrylate groups, validating the successful synthesis of gelMA. Furthermore, the increased signal of methyl groups at 1.8 ppm and the decreased signal of amine groups at 2.9 ppm in gelMA compared to gelatin indicate the transformation of amine groups into methacrylate groups. The observed peaks were also in agreement with values reported previously in the literature [46,47].

Moreover, the degree of amine substitution in gelMA can be controlled during the synthesis process by adjusting the amount of methacrylic acid anhydride and reaction time. To quantify the degree of substitution, the amine groups present in gelMA were quantified through fluoraldehyde analysis, as these groups in gelatin undergo substitution by methacrylate groups. The quantitative analysis revealed that the synthesized gelMA represented a methacrylate group substitution level of 76 ± 5%.

**Figure 2:**
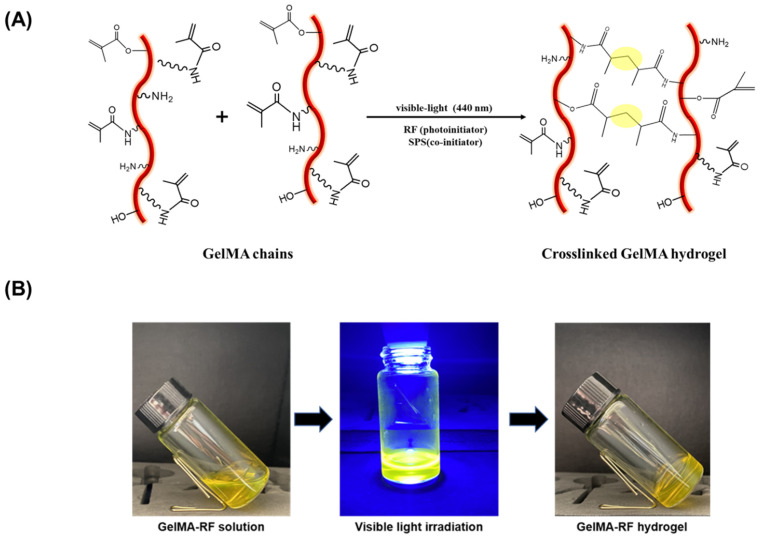
(**A**) Schematic illustration of gelMA hydrogel photo-crosslinking reaction (**B**) Image of the formation of gelMA-RF hydrogel under visible light for 30 s.

When visible light is applied to gelMA with the addition of RF, gelation occurs. RF serves as a Type II photoinitiator, acting as a cationic donor to initiate the photopolymerization reaction in the gelMA hydrogel, as shown in Figure 2A [48]. Sodium persulfate is employed as a co-initiator to facilitate this reaction. Under visible light exposure, RF becomes excited and forms radicals with methacrylic anhydride. This initiates a crosslinking reaction between the vinyl moieties in the methacrylic groups of gelMA molecules, leading to the formation of the hydrogel.

The sol–gel transition of the conjugate was confirmed by a tube inversion method [42]. At 37 °C, the gelMA solution exhibited a flowing characteristic and demonstrated gelation when exposed to visible light for 30 s (Figure 2B).

Injectable hydrogel systems utilizing biomaterials provide notable benefits in overcoming limitations in minimally invasive surgical procedures [49]. GelMA hydrogels can easily adapt to defect shapes and serve as an injectable system for filling cartilage defect areas.

**Figure 3:**
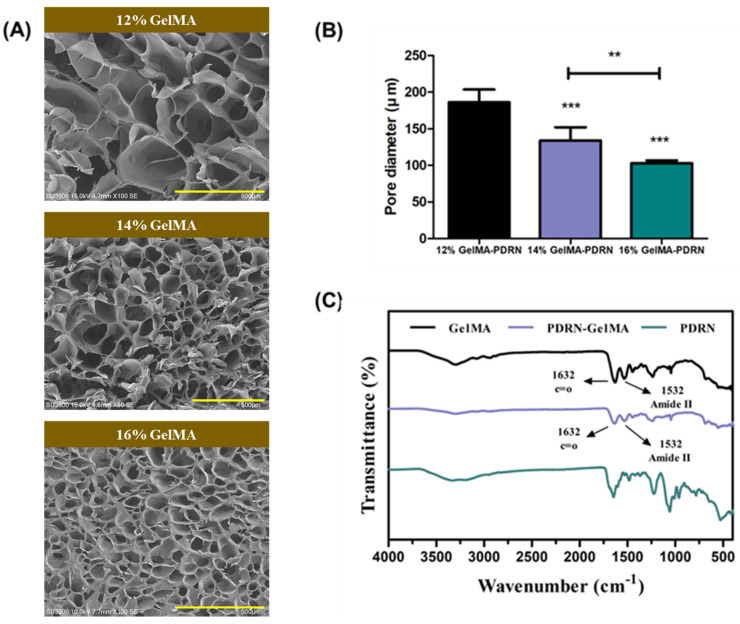
(**A**) SEM images of cross-sections of gelMA hydrogel and gelMA-PDRN hydrogel, (**B**) The average pore size counted by ImageJ based on SEM images (scale bar = 500 μm) (mean ± SD, n = 3, ** *p* < 0.01, *** *p* < 0.001) (**C**) FT-IR spectra of PDRN only, gelMA, and gelMA-PDRN hydrogel.

The characterization of gelMA hydrogels was performed after loading PDRN. The freeze-dried gelMA hydrogel exhibited a porous structure when observed using scanning electron microscopy (SEM, Figure 3A). Hydrogel’s porosity and pore size are crucial design elements in tissue engineering, as they play a significant role in regulating substance exchange between the external environment and the internal environment of the hydrogel, as well as influencing cellular behaviors. These factors are closely associated with tissue formation, making them essential considerations in the field of tissue engineering [50,51]. With different compositions, 12%, 14%, and 16% gelMA exhibited pore sizes of 178.27 ± 2.64 μm, 133.27 ± 2.10 μm, and 103.11 ± 3.38 μm, respectively (Figure 3B). The pore sizes in all groups fell within the range of 100–200 μm, indicating sizes conducive to the attachment and proliferation of chondrocytes. The variation in porous structures revealed that as the gelMA content increased, the chemical crosslinking density increased, enhancing the interconnectivity between each matrix. This resulted in a gradual reduction in pore size, demonstrating the correlation between chemical crosslinking density and pore size decrease [52].

To ensure stable loading of PDRN, it was essential to verify the absence of any chemical bonding between PDRN and gelMA. Therefore, Figure 3C shows the FT-IR spectra of PDRN alone, gelMA, and PDRN-gelMA. GelMA hydrogel exhibits strong characteristic peaks at 1632 cm^−1^ (C=O stretching group) and 1532 cm^−1^ (amide II). Identical peaks are observed in gelMA hydrogel loaded with PDRN, indicating that there was no specific chemical bond formation between the gelMA polymer and PDRN.

### 2.2. GelMA Hydrogel Mechanical Properties

**Figure 4:**
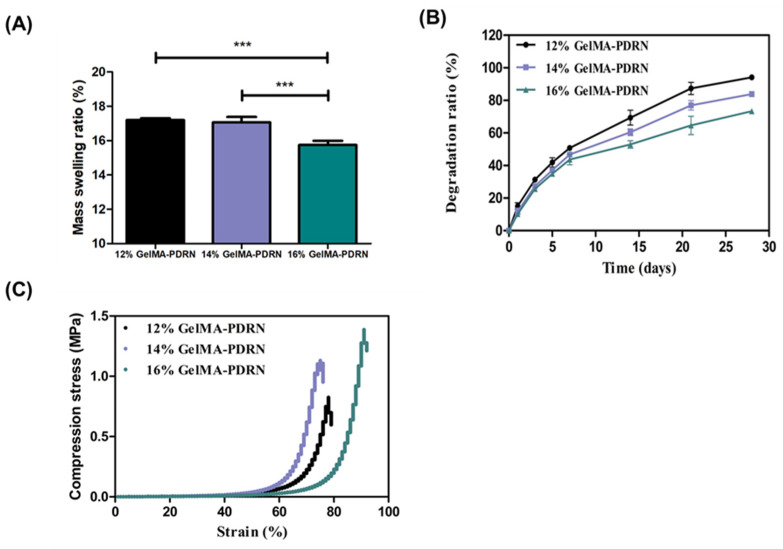
Mechanical properties of gelMA-PDRN hydrogels (**A**) Mass swelling of gelMA-PDRN hydrogels (mean ± SD, n = 6) (**B**) Degradation ratio analyzed for 28 days (mean ± SD, n = 6) (**C**) Strain–stress curve of gelMA-PDRN hydrogel (mean ± SD, n = 3) (*** *p* < 0.001).

The mass swelling ratio of hydrogels is a crucial parameter that quantifies the degree to which the support material absorbs water from the surroundings, thereby impacting a wide range of applications, including regulating the diffusion between the external environment and internal molecules, as well as facilitating cell movement and proliferation. The mass swelling ratio exhibits considerable variation depending on the pore size of chemically crosslinked gels and the interaction between the hydrogel matrix and the solvent. Hydrophilic hydrogels have functional groups, such as hydroxyl (-OH), carboxyl (-COOH), and amino (-NH_2_) groups, that can form hydrogen bonds with water molecules. This increases the ability of the hydrogel to absorb and retain water. Due to their strong affinity for water, hydrophilic hydrogels tend to swell significantly, resulting in a high mass swelling ratio. We analyzed the swelling ratio of hydrogels with varying polymer concentrations shown in Figure 4A. The swelling ratios of 12% gelMA-PDRN, 14% gelMA-PDRN, and 16% gelMA-PDRN hydrogels were determined to be 17.20 ± 0.09%, 16.90 ± 0.04%, and 15.64 ± 0.12%, respectively. This yielded results similar to the mass swelling ratio observed in previously studied hydrophilic polymers, such as PVA and PEG hydrogels [53,54]. An inverse correlation was observed between polymer content and mass swelling ratio. The observed result can be explained by the increased crosslinking density among polymer chains, which occurs when the concentration of the polymer in the hydrogel increases.

The rate at which the scaffold degrades has an impact on the process of tissue regeneration [55]. Gelatin-based hydrogels, such as gelMA, possess the characteristic of being degraded in the presence of collagenase. To analyze the degradation rate of gelMA hydrogels, they were cultured in PBS containing collagenase type II. The degradation rate was subsequently analyzed under these conditions [56]. As shown in Figure 4B, it was determined that each hydrogel showed incomplete degradation during the 28-day. The degradation rates for gelMA-PDRN hydrogels with concentrations of 12%, 14%, and 16% were 94%, 83%, and 73%, respectively, over 28 days. It can be explained by the increased crosslinking density resulting from increased polymer concentration.

When designing the scaffold for cartilage tissue engineering, the cartilage, which serves as a tissue supporting the body’s load, is a crucial factor to be considered. To assess sufficient support for the engineered hydrogels as scaffolds for cartilage tissue engineering, compression stress was evaluated in Figure 4C. The maximum stress values for 12% gelMA-PDRN, 14% gelMA-PDRN, and 16% gelMA-PDRN hydrogels were observed to be 0.75 MPa, 1.10 MPa, and 1.38 MPa, respectively. As the gelMA content increases, the mechanical properties of the hydrogel improve, confirming that higher mechanical properties are achieved with an increase in the cross-linking density of gelMA. Furthermore, the significantly increased mechanical properties, compared to the previously studied gelatin-based hydrogels, suggest its potential application as a scaffold for cartilage tissue engineering [57,58].

### 2.3. PDRN Release Profile in gelMA Hydrogels

**Figure 5:**
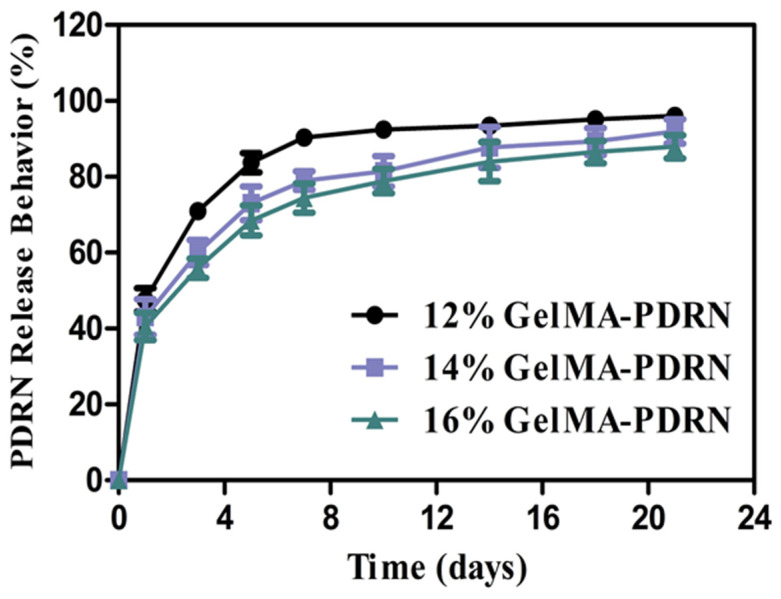
PDRN release profile of the gelMA-PDRN hydrogels with different gelMA concentrations (mean ± SD, n = 6).

We aimed to measure the amount of PDRN released in gelMA hydrogels in PBS containing collagenase type II in Figure 5. The measurements were conducted based on absorbance at a wavelength of 260 nm, and the release behavior of PDRN was expressed as cumulative percentage release. In all groups, over 40% of the drug was released due to the initial burst release, and the group with the lowest gelMA content, the 12% gelMA/PDRN group, exhibited over 90% drug release after 7 days. For the 14% gelMA-PDRN and 16% gelMA-PDRN groups, a sustained drug release behavior was observed until day 21. The degree of physical interconnection between PDRN and gelMA had a direct impact on the release characteristics of PDRN from the hydrogel carrier.

The size of PDRN exhibits a diverse range, spanning from 100 to 1200 nm [9]. Due to this varied size distribution, relatively small-sized PDRN can be released through the mesh spaces of the hydrogel, while PDRN with a size exceeding 1000 nm is confirmed to be encapsulated stably within the mesh spaces, particularly as the concentration of gelMA increases.

### 2.4. Cytotoxicity Test of Hydrogels

**Figure 6:**
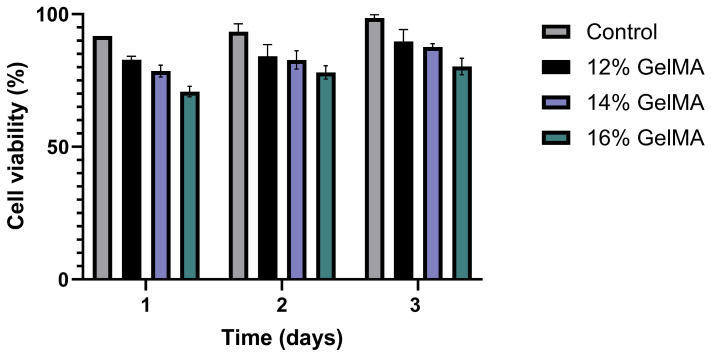
Cell viability of examined GelMA-PDRN hydrogels by MTT assay for 3 days (mean ± SD, n = 6).

The evaluation of hydrogel cytotoxicity is a fundamental assessment method in the field of tissue engineering to confirm the biocompatibility of materials. To assess the cytotoxicity of the hydrogel, NIH-3T3 cells were cultured for 1, 2, and 3 days, followed by performing an MTT assay (Figure 6). The MTT assay was used to determine cell viability based on an untreated control group as a reference for hydrogel immersion. After 3 days of culture, MTT results indicated cell survival rates of 88.10 ± 1.43%, 86.13 ± 0.89%, and 80.47 ± 0.81% for 12% gelMA-PDRN, 14% gelMA-PDRN, and 16% gelMA-PDRN hydrogels, respectively. All groups showed a cell survival rate of over 80%. However, with an increase in gelMA content, cell toxicity increased, with respective cell survival rates of 82.61 ± 0.54%, 77.03 ± 0.77%, and 68.00 ± 0.52% observed after 1 day of culture, indicating higher initial cell toxicity for 16% gelMA-PDRN. Hence, after evaluating the materials in our study, we determined that the composition of 14% gelMA-PDRN was the most suitable for delivering PDRN due to its excellent biocompatibility and consistent release behavior.

### 2.5. Glycosaminoglycan (GAG) Activity

**Figure 7:**
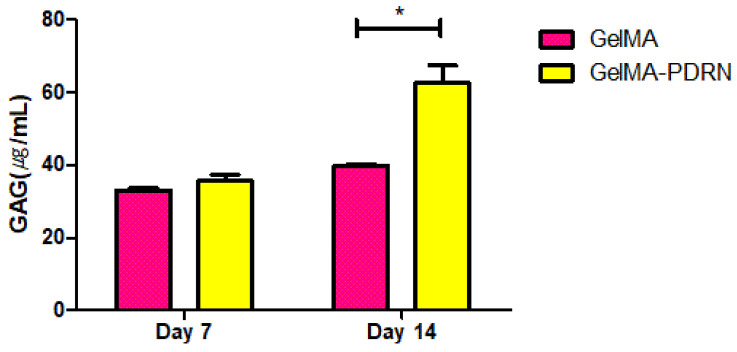
Biochemical characterization with GAG quantitative analysis (mean ± SD, n = 6, * *p* < 0.05).

Glycosaminoglycan (GAG) is an essential extracellular matrix (ECM) component of cartilage, and its formation is directly related to cartilage development. To indirectly assess the impact of PDRN released in hydrogel on cartilage formation, the quantification of formed GAG was analyzed after 7 and 14 days of culture in Figure 7. It has been observed that the group containing PDRN in gelMA exhibited a higher GAG content on the 14 days of cell culture. According to previous research, it has been observed that in the PDRN-treated group, the gene MMP-13 associated with cartilage destruction is downregulated. In this context, the sustained release of PDRN is considered to minimize the negative impact on chondrocytes and play a role in maintaining a higher expression of GAG [11].

### 2.6. Evaluation of Cartilage-Specific Genes Expression

Because substrate-producing cells in the cartilage aid in the development of new tissue, maintaining the chondrocyte phenotype is critical for the healing of cartilage tissue in vivo [59].

**Figure 8:**
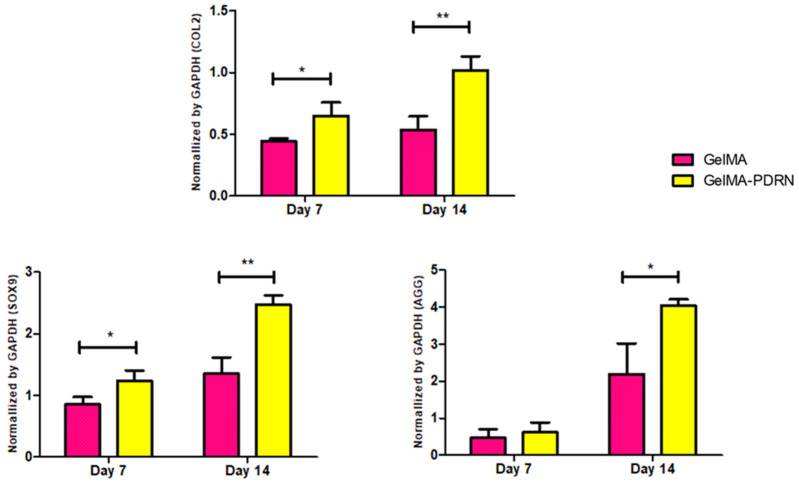
Cartilage specific gene expression evaluated by RT-PCR with COL2, SOX9, and AGG normalized by GAPDH (mean ± SD, n = 6, * *p* < 0.05, ** *p* < 0.01).

To assess the gene expression of cartilage-specific genes COL2, SOX9, and AGG for cartilage tissue regeneration, RT-PCR analysis was conducted after 7 and 14 days of cultivation. GAPDH was used as a housekeeping gene (Figure 8). COL2 is a key component constituting cartilage tissue, SOX9 is involved in the differentiation and formation of chondrocytes, and AGG is an essential component for the structure of cartilage and joint function. On 14 days of culture, COL2, SOX9, and AGG expressions were 1.9-, 1.8-, and 1.8-fold higher in gelMA-PDRN compared to only gelMA, respectively. There is still no obvious mechanism for maintaining avascularity in articular cartilage. Nonetheless, it has been proposed that endogenous angiogenesis inhibitors are highly concentrated in hyaline cartilage [60]. As known, the increase in angiogenesis by PDRN may have negative effects on chondrocytes. However, contrary to what was previously known, it can be speculated that the angiogenic effects of PDRN itself do not negatively impact chondrocytes. By confirming a significant increase in the expression of cartilage-specific genes, PDRN could be considered as a novel therapeutic approach for cartilage-related conditions such as osteoarthritis [61].

### 2.7. Histology Assessment

**Figure 9:**
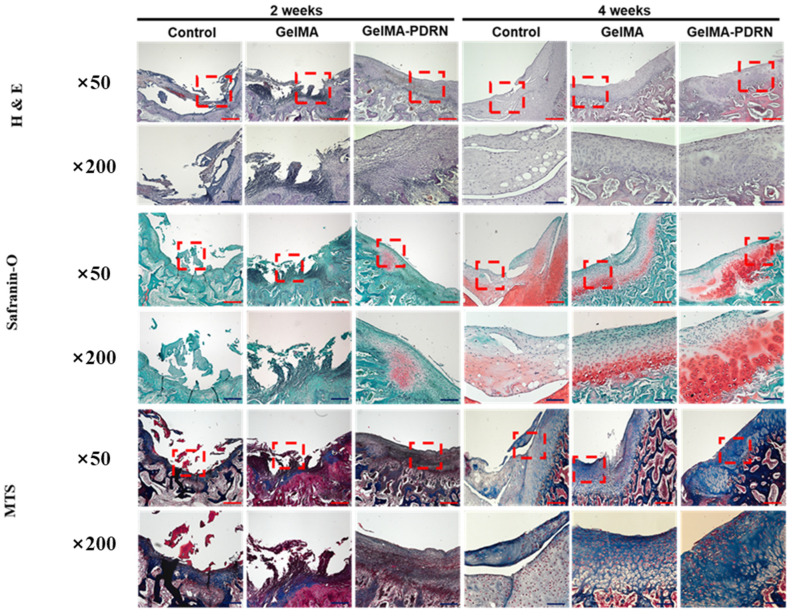
Histology sections of all groups of the control, GelMA, and GelMA-PDRN groups were displayed at 2 and 4 weeks at 50× (scale bars = red 200 μm) and 200× (scale bars = blue 40 μm) magnifications with H&E, Safranin O, and MTS staining.

To assess tissue regeneration through gelMA and gelMA-PDRN transplantation for articular cartilage defects in vivo, tissue samples at 2 and 4 weeks were subjected to H&E, Safranin-O staining, and Masson’s trichrome stain (MTS) analysis (Figure 9).

Observations of cellular nuclei and cytoplasm, revealing tissue microstructure and cell types, were made through H&E staining. In the 2-week group, irregular and unrepaired tissue morphology was observed in the control group without hydrogel transplantation and gelMA hydrogel. In contrast, gelMA-PDRN exhibited a regenerated smooth tissue surface. In the 4-week group, the control group displayed irregular tissue surface due to numerous fibroblasts hindering proper cartilage tissue regeneration. In contrast, gelMA and gelMA-PDRN hydrogels formed a smooth tissue surface and organized layers of chondrocytes. This suggests that gelMA hydrogel creates an effective microenvironment for cell proliferation and growth, positively influencing tissue formation. Moreover, gelMA hydrogel containing PDRN promoted chondrocyte proliferation and growth, leading to effective cartilage tissue regeneration.

Safranin-O staining, highlighting glycosaminoglycans in red, was performed to confirm cartilage formation. In the 2-week group, the control group and gelMA hydrogel showed minimal red-stained chondrocyte layers, while gelMA-PDRN displayed some red-stained chondrocyte layers. In the 4-week group, the control group exhibited a faint red layer, whereas gelMA and gelMA-PDRN hydrogels showed distinct red layers, indicating active chondrocyte proliferation. GelMA-PDRN hydrogel displayed a thicker red layer, suggesting a beneficial effect of PDRN on chondrocyte proliferation and growth [62].

Lastly, Masson’s trichrome stain was conducted to confirm extracellular matrix (ECM) generation through collagen formation. In the 2-week group, the defect site exhibited minimal blue-stained collagen. In the 4-week group, the control group showed insufficient collagen formation, and numerous fibroblasts indicated inadequate cartilage regeneration. In contrast, gelMA and gelMA-PDRN hydrogels demonstrated reduced fibrous content, with uniformly increased blue-stained collagen, indicating active cartilage regeneration. Notably, gelMA-PDRN hydrogel exhibited the most vigorous collagen formation, highlighting the superior regenerative effect of PDRN on cartilage.

Overall, histological staining results suggest that the microstructure of gelMA hydrogel creates a favorable microenvironment for cell attachment, growth, and proliferation, resembling articular cartilage. Additionally, gelMA-PDRN reduces inflammation and degradation of essential proteoglycans and collagen necessary for chondrocyte formation, promoting chondrocyte proliferation and growth, thus facilitating cartilage tissue regeneration.

## 3. Conclusions

The aim of this study was to develop a visible light photo-crosslinked gelatin Methacrylate (gelMA) hydrogel that induces sustained release of Polydeoxyribonucleotide (PDRN) for cartilage regeneration. Although there is still insufficient evidence to definitively explain the positive effects of PDRN on cartilage regeneration, this research demonstrated positive outcomes in both in vitro and in vivo settings. The gelMA hydrogel, synthesized through visible-light-induced crosslinking with Riboflavin 5′-phosphate sodium (RF) as a photoinitiator, exhibited suitable physicochemical properties for cartilage regeneration. The incorporation of PDRN addressed drawbacks in traditional injection methods and contributed to enhanced hydrogel characteristics.

While this study provides valuable insights into developing an effective hydrogel system for cartilage regeneration, certain limitations and areas for improvement should be acknowledged. Future research could explore the optimization of gelMA-PDRN formulations, investigate long-term effects, and delve into the molecular mechanisms underlying the observed regenerative outcomes. Furthermore, assessing the clinical applicability and conducting additional validation in large animal models will be crucial steps in establishing the translational potential of gelMA-PDRN hydrogel in the field of cartilage tissue engineering.

## 4. Materials and Methods

### 4.1. Reagents and Materials

Gelatin, type A (porcine skin, 300 g Bloom), methacrylic anhydride, Riboflavin 5′-phosphate sodium, and Sodium persulfate were purchased from Sigma-Aldrich (St. Louis, MO, USA). Polydeoxyribonucleotide (PDRN) was obtained from Humedix (Seongnam, Gyeonggi-do, Republic of Korea). MSC SFM, Fetal Bovine Serum (FBS), Penicillin-Streptomycin (PS), and Phosphate Buffered Saline (PBS) were procured from Gibco (Waltham, MA, USA).

### 4.2. Synthesis Process of GelMA

GelMA used in this study was prepared according to a previously reported method [45]. Briefly, gelatin was heated and stirred at 50 °C in deionized water until completely dissolved at a concentration of 10% (*w*/*v*) for 1 h. Methacrylic anhydride (gelatin:methacrylic anhydride = 1:0.6) was then added dropwise with rapid stirring, and the reaction proceeded for 3 h. Unreacted methacrylic anhydride was removed by centrifugation at 350× *g* for 3 min, and the supernatant was collected. The collected supernatant was diluted with deionized water (supernatant:deionized water = 1:2) at 40 °C to stop the reaction. The resulting solution was dialyzed using a 12~14 kDa membrane against deionized water at 40 °C for 1 week. Subsequently, 1 M NaHCO_3_ was added to adjust the solution pH to 7.4. After removing residual impurities through a 0.2 μm syringe filter, the solution was cooled at −80 °C and freeze-dried for 1 week to completely remove moisture. The dried gelMA was stored at −20 °C until use.

### 4.3. Preparation of PDRN-Loaded gelMA Hydrogel

The synthesized gelMA was added to phosphate-buffered saline (PBS) at concentrations of 12%, 14%, and 16% (*w*/*v*) and stirred until completely dissolved at 50 °C. PDRN was then added to the gelMA solution at a concentration of 100 μg/mL. Subsequently, 0.5 mM riboflavin and 20 mM sodium persulfate were added, and the mixture was stirred thoroughly until well-mixed. The resulting solution was poured into silicone molds, and the hydrogel was manufactured through photo-crosslinking by exposing it to visible light (>1400 mW/cm^2^; Foshan Keyuan Medical Equipment Co., Ltd., Foshan, China) for 30 s.

### 4.4. Evaluation of the Physicochemical Properties of gelMA Hydrogel

#### 4.4.1. ^1^H-Nuclear Magnetic Resonance

The chemical composition and structure of the synthesized GelMA were analyzed by dissolving gelatin and GelMA in deuterium oxide (D_2_O) at 35 °C, using proton nuclear magnetic resonance spectroscopy (^1^H NMR, 500 MHz, JEOL FT-NMR Spectrometer, Peabody, MA, USA). The spectra were recorded, and chemical shifts were referenced to the residual solvent peak of D_2_O at 4.79 ppm.

#### 4.4.2. Determination of Degree of Functionalization of gelMA

To quantitatively determine the degree of methacrylation in the amine groups of gelatin, a fluoraldehyde assay was conducted. gelMA at 0.5 mg/mL and gelatin at concentrations of 0.02, 0.1, 0.5, and 1 mg/mL were completely dissolved in PBS at 50 °C. The solutions were then cooled to room temperature. A solution of phthalaldehyde (Phthalaldehyde) was prepared (600 µL), and it was mixed with the prepared gelMA, gelatin solutions, and phosphate-buffered saline (PBS) (300 µL) for 1 min. The fluorescence intensity of each solution was measured at a 450 nm wavelength using a microplate reader (Synergy MX, BioTek, Winooski, VT, USA). The fluorescence intensity of the PBS group was subtracted from the average fluorescence intensity of each group. A standard curve was generated based on the mean fluorescence intensity according to gelatin concentration, and using this, the methacrylation degree of gelMA was calculated based on the gelMA fluorescence intensity and gelatin content (*X* mg/mL) according to Formula (1)

(1) $$\mathrm{Degree}\;\mathrm{of}\;\mathrm{functionalization}=\frac{\left(0.5-X\right)}{0.5}\times 100\;(\%)$$

#### 4.4.3. Fourier Transform Infrared Spectroscopy (FT-IR)

Fourier Transform Infrared Spectroscopy (FT-IR) was employed to analyze the chemical structure and functional groups of the synthesized GelMA hydrogels. The samples were prepared by drying the hydrogels and grinding them into a fine powder. The FT-IR spectra were obtained using a Thermo Scientific Nicolet iS10 FT-IR spectrometer (Thermo Fisher Scientific, Waltham, MA, USA) equipped with a Zinc Selenide (ZnSe) prism. The measurements were conducted in the range of 4000–400 cm^−1^ with a resolution of 4 cm^−1^ and an average of 32 scans per sample.

#### 4.4.4. Morphological Analysis

For morphological analysis, including the evaluation of the porous surface and pore size of the hydrogel, scanning electron microscopy (Variable Pressure SEM, Hitachi, Chiyoda, Tokyo, Japan) was employed. The samples were subjected to freeze-drying for three days, with sequential durations of 4 h at 4 °C, 4 h at −20 °C, and 12 h at −80 °C, ensuring complete removal of moisture. Prepared samples were cross-sectioned to preserve pore integrity, affixed to sample mounts with carbon tape, coated with platinum, and examined for morphological structure. The pore diameter was analyzed using ImageJ software (ImageJ 1.50i, National Institutes of Health, Bethesda, MD, USA).

#### 4.4.5. Water Content Analysis of gelMA Hydrogels

The swelling ratio of the hydrogel over time was measured. The initial weight (w_d_) was recorded, and each hydrogel was immersed in 1 mL of PBS at 37 °C for 24 h. At various measurement times (5, 10, 15, 30 min, 1, 2, 4, 8, 16, and 24 h), the hydrogel was gently wiped with a KimWipe to remove excess liquid, and the swollen weight (w_s_) was recorded. The mass swelling ratio was calculated according to Formula (2)

(2) $$\mathrm{Swelling}\;\mathrm{ratio}\;(\%)=\frac{w_{s}-w_{d}}{w_{d}}\times 100\;(\%)$$

#### 4.4.6. Degradation Studies

The enzymatic degradability of the hydrogel was assessed. After freeze-drying, the initial dried weight was measured, and the hydrogel was then immersed in a 500 µL solution of diluted Collagenase Type II (Worthington Biochemical, Lakewood, OH, USA) in PBS, stored at 37 °C. To maintain enzyme activity, the solution was replaced every two days. At each measurement time point (1, 3, 5, 7, 14, 21, and 28 days), the hydrogel was collected, washed three times with PBS, freeze-dried, and the weight (Mass at time point) was measured. The degradation rate was calculated according to Formula (3)

(3) $$\mathrm{Degradation}\;\mathrm{ratio}\;(\%)=\;\frac{Initial\;dried\;weight-Weight\;at\;time\;point}{Initial\;dried\;weight}\times 100\;(\%)$$

#### 4.4.7. Compression Test of gelMA Hydrogels

To measure the mechanical strength, the prepared hydrogel was immersed in PBS at 37 °C for 24 h to simulate a biologically relevant environment. The diameter and height of the hydrogel were measured using calipers (Mitutoyo, Kawasaki, Japan). Compression strength assessment was conducted using a materials testing system (TMS-Pro, Food Technology Corporation, Livermore, CA, USA). The measurements were performed under the conditions of a 40 N load cell and a testing speed of 2.0 mm/min [63].

#### 4.4.8. Release Profile of PDRN

To analyze the release behavior of PDRN within the hydrogel, the manufactured hydrogel was immersed in a solution of diluted Collagenase Type II in PBS and stored at 37 °C. At each measurement time point (1, 3, 5, 7, 10, 14, 18, and 21 days), aliquots of the solution were collected, and fresh solution was replenished. The collected extracts were filtered using a 0.45 μm syringe filter to remove residues, and the absorbance at 260 nm was measured using a microplate reader (Synergy MX, BioTek, USA).

### 4.5. Evaluation of the Physicochemical Properties of GelMA Hydrogel

#### 4.5.1. Cell Culture

The embryonic mouse fibroblast cell line NIH3T3 (National Institutes of Health, KCLB21658) used in the experiments was cultured in RPMI medium (Gibco, Waltham, MA, USA) supplemented with 10% fetal bovine serum (FBS, Gibco, USA) and 1% penicillin/streptomycin (PS, Gibco, USA).

Rabbit chondrocytes (rChons) were isolated from the knee joints of 4-week-old female New Zealand white rabbits obtained from Hanil Laboratory Animal Center, Korea. The rChons were cultured in Dulbecco’s Modified Eagle Medium: Nutrient Mixture F-12 (DMEM/F12, Gibco, USA) supplemented with 10% FBS and 1% PS. All cells were cultured in a standard cell culture incubator (5% CO_2_, 37 °C) with media replacement every 3 days.

#### 4.5.2. Cytotoxicity Test

For the cytotoxicity assessment of hydrogels, NIH/3T3 cells were seeded in 24-well plates at a density of 2 × 10^4^ cells/well using RPMI cell culture medium and incubated in a cell culture incubator (5% CO_2_, 37 °C) for 24 h. gelMA-PDRN hydrogel samples were then applied and cultured for 1, 2, and 3 days. At specific time points, the hydrogel was removed, and the cells were treated with MTT (3-[4,5-Dimethyl-2-thiazolyl]-2,5-diphenyl-2H-tetrazolium bromide; thiazolyl Blue, Amresco, Solon, OH, USA) solution, followed by a 3 h incubation under conditions of 5% CO_2_, 37 °C. The supernatant was removed, and dimethyl sulfoxide (DMSO; Samchun Chemical, Incheon, Republic of Korea) was added to dissolve the formazan crystals completely. Absorbance was measured at 570nm using a microplate reader (Synergy MX, Biotek, Vernusky, VT, USA). All groups were normalized to the control group (RPMI medium), and cell viability was calculated using the following Formula (4)

(4) $$\mathrm{Cell}\;\mathrm{viability}\;(\%)=\frac{{OD}_{Sample}}{{OD}_{Control}}\;\times \;100\;(\%)$$

#### 4.5.3. Glycosaminoglycan (GAG) Assay

For rChons, cells were seeded in a 24-well plate at a density of 2 × 10^4^ cells/well using DMEM/F12 cell culture medium. The cells were then cultured with gelMA and gelMA-PDRN for 7 and 14 days under conditions of 37 °C and 5% CO_2_. The cell culture medium was replaced every 3 days. Subsequently, all samples were washed three times with PBS after removing the cell culture medium and hydrogel. The cells were then lysed by adding RNAiso Plus (Takara Bio, Shiga, Japan), and the cell lysate was collected in a 1.5 mL Eppendorf tube (EP tube, Eppendorf, Hamburg, Germany) to prepare the cell lysate solution.

Quantitative analysis was performed using the GAG quantitative kit (Glycosaminoglycan Assay Kit, Chondrex, Woodinville, WA, USA). In brief, the prepared cell lysate was centrifuged at 14,000 rpm at 4 °C for 10 min to achieve sufficient separation. The supernatant was then vigorously vortexed to facilitate GAG separation. The resulting solution was transferred to a 96-well plate, and GAG dye solution was added. After incubating at room temperature for 5 min, the absorbance was measured at 525 nm using a microplate reader.

#### 4.5.4. Evaluation of Cartilage-Specific Genes Expression

To assess the expression of cartilage-specific genes, Real-Time Polymerase Chain Reaction (RT-PCR) was conducted. Rabbit chondrocytes (rChons) were seeded in a 24-well plate at a density of 2 × 10^4^ cells/well and cultured with gelMA and gelMA-PDRN. At specific time points, the culture medium and hydrogel were removed, and the cells were washed three times with PBS. For cell lysis, RNAiso Plus was added, and the dissolved cells were collected in a 1.5 mL Eppendorf tube, followed by mixing with chloroform (Samchun Chemicals, Republic of Korea). Centrifugation at 13,000 rpm, 4 °C, for 15 min separated the transparent upper phase. The upper phase was mixed with isopropanol (Sigma-Aldrich, USA) and stored at −20 °C for 24 h. After centrifugation at 13,000 rpm, 4 °C, for 15 min, the upper phase was removed. Ethanol (75%, 25% DNase/RNase-Free Distilled Water) was added, followed by centrifugation at 13,000 rpm, 4 °C, for 5 min, and the upper phase was removed. This process was repeated twice. The extracted mRNA was diluted with DNase/RNase-Free Distilled Water (Invitrogen, Carlsbad, CA, USA), and its concentration was quantified using a BioSpectrophotometer (Eppendorf, Hamburg, Germany). cDNA synthesis was performed using TopscriptTM RT DryMIX (dT18plus, Enzynomics, Seoul, Republic of Korea) according to the in-house protocol and a PCR Thermal Cycler (Takara Bio Inc., Shiga, Japan). The synthesized cDNA was mixed with primers for each gene and SYBRTM Green PCR Master Mix (Applied Biosystems, Foster City, CA, USA), and real-time PCR was conducted using the StepOnePlus Real-Time PCR system (Applied Biosystems, USA). The gene expression levels of Collagen Type II (COL2), Aggrecan (AGG), and Transcription factor SOX-9 (SOX9) were evaluated, and the transcription levels of all genes were normalized using the GAPDH gene (Table 2).

### 4.6. In Vivo Cartilage Defect Model and Implantation Hydrogels

All animal experiments were conducted in accordance with the guidelines and approval of the Institutional Animal Care and Use Committee at Jeonbuk National University (JBNU 2022-067). Prior to the experiments, all surgical instruments used were subjected to sterilization using a high-temperature and high-pressure autoclave, and the surgeries were performed under general inhalation anesthesia. To assess the in vivo response to cartilage defects in gelMA and gelMA-PDRN hydrogels, a rabbit cartilage defect model was created using 6-week-old male New Zealand white rabbits (Hanil Laboratory Animal Center, Yongin, Gyeonggi-do, Republic of Korea). At first, the area where the surgery was to be performed was sterilized using a solution containing 70% ethanol and povidone-iodine. Afterwards, a 6 mm biopsy punch manufactured by Kai Industry in Japan was employed to generate a circular defect on the cartilage of the rabbit’s knee. A circular cartilage defect, measuring 6 mm in diameter and 2 mm in height (equivalent to the average cartilage thickness), was created using a 1 mm drill. The gelMA solution and gelMA-PDRN solution were injected into the cartilage defect sites, ensuring that the volume of the solutions matched the size of the defects. Gelation was initiated by exposure to visible light for 30 s. The rabbits were sacrificed 2 and 4 weeks after the procedure to obtain tissue samples.

### 4.7. Histological Analysis

At each time point, cartilage samples were extracted and fixed in 10% formalin solution (Formalin solution, Sigma-Aldrich). The samples were treated with Decalcifying Solution-Lite (Sigma-Aldrich) to remove calcium and then dehydrated using a sequence of ethanol solutions with varying concentrations, starting from low and gradually increasing to high. Afterward, the samples underwent treatment with xylene (Xylene, Sigma-Aldrich, USA). Subsequently, the specimens were submerged in paraffin to guarantee adequate paraffin infiltration and subsequently embedded in paraffin to create tissue blocks. And prepared paraffin blocks were cut into sections with a thickness of 10 μm, and these sections were then attached to slides that had been coated with poly-L-lysine. Following that, the paraffin was eliminated at a temperature of 60 °C, and any residual paraffin on the samples was removed using xylene. Gradually decreasing quantities of ethanol were used to induce a dehydration process. The staining techniques, including H&E, MTS, and safranin-O, were carried out in accordance with the established methodology. An optical microscope (Leica DM2500, Leica Microsystems GmbH, Wetzlar, Germany) was used to analyze the stained samples.

### 4.8. Statistics

All data results are presented as mean ± standard deviation (SD). GraphPad Prism 5.0 software (GraphPad Software, La Jolla, CA, USA) was utilized for statistical analysis, and verification was performed using a two-way ANOVA test. The significance levels were considered as follows: *p* < 0.05 (*), *p* < 0.01 (**), and *p* < 0.001 (***).

## Figures and Tables

**Table 2:** Sequences of primers used in quantitative real-time reverse transcription polymerase chain reaction.

Gene	Forward (F) and Reverse (R) Primer Sequence (5′→3′)	
GAPDH	F	CGACTTCAACAGCGACACTCAC
	R	CCCTGTTGCTGTAGCCAAATTC
COL2	F	GCCACTGGATTCCCTGGAGCT
	R	TCTTGCTGCTCCACCAGTTC
AGG	F	GCCACTGGATTCCCTGGAGCT
	R	TCTGCAGCAGTTGATTCTGATT
SOX9	F	ACGGAGCAGACGCACATCTC
	R	CTTCTCGCTCTCGTTCAGAAGT

## Data Availability

The data presented in this study are available on request from the corresponding author.
